# Development of SCAR markers for rapid and specific detection of *Pseudomonas syringae* pv. *morsprunorum* races 1 and 2, using conventional and real-time PCR

**DOI:** 10.1007/s00253-016-7295-0

**Published:** 2016-02-01

**Authors:** Monika Kałużna, Pedro Albuquerque, Fernando Tavares, Piotr Sobiczewski, Joanna Puławska

**Affiliations:** 1 Research Institute of Horticulture, Konstytucji 3 Maja 1/3, 96-100 Skierniewice, Poland; 2 Centro de Investigação em Biodiversidade e Recursos Genéticos (CIBIO), InBIO, Laboratório Associado, Universidade do Porto, Campus Agrário de Vairão, 4485-661 Vairão, Portugal; 3 Faculdade de Ciencias, Departamento de Biologia, Universidade do Porto, Edifício FC4, Via Panoramica No. 36, 4150-564 Porto, Portugal

**Keywords:** Dot blot hybridisation, Stone fruit tree pathogens, PCR MP, SCAR primers, Real-time PCR

## Abstract

Specific primers were developed to detect the causal agent of stone fruit bacterial canker using conventional and real-time polymerase chain reaction (PCR) methods. PCR melting profile (PCR MP) used for analysis of diversity of *Pseudomonas syringae* strains, allowed to pinpoint the amplified fragments specific for *P. syringae* pv. *morsprunorum* race 1 (*Psm*1) and race 2 (*Psm*2), which were sequenced. Using obtained data, specific sequence characterised amplified region (SCAR) primers were designed. Conventional and real-time PCRs, using genomic DNA isolated from different bacterial strains belonging to the *Pseudomonas* genus, confirmed the specificity of selected primers. Additionally, the specificity of the selected DNA regions for *Psm*1 and *Psm*2 was confirmed by dot blot hybridisation. Conventional and real-time PCR assays enabled accurate detection of *Psm*1 and *Psm*2 in pure cultures and in plant material. For conventional PCR, the detection limits were the order of magnitude ~10^0^ cfu/reaction for *Psm*1 and 10^1^ cfu/reaction for *Psm*2 in pure cultures, while in plant material were 10^0^–10^1^ cfu/reaction using primers for *Psm*1 and 3 × 10^2^ cfu/reaction using primers for *Psm*2. Real-time PCR assays with SYBR Green I showed a higher limit of detection (LOD) − 10^0^ cfu/reaction in both pure culture and in plant material for each primer pairs designed, which corresponds to 30–100 and 10–50 fg of DNA of *Psm*1 and *Psm*2, respectively. To our knowledge, this is the first PCR-based method for detection of the causal agents of bacterial canker of stone fruit trees.

## Introduction

Bacterial canker of fruit trees occurs in stone fruit growing areas all over the world (Agrios 2005). In Poland, the disease incidence on stone fruit trees orchards is observed every year with different intensity and is becoming more economically significant. Moreover, in the last vegetative seasons, bacterial canker was dangerous not only to stone fruit trees, but also to apple and pear trees. The causal agents of the disease belong to the polyphagous *Pseudomonas syringae* species, able to infect more than 180 plant species, both annual and perennial, including fruit trees, ornamental plants and vegetables. *P. syringae* affects all organs of the aboveground parts of trees (i.e. the branches and main trunk as well as buds, blossoms, leaves and fruits), which causes reduction of yield and sometimes leads to death of the trees.

*P. syringae* is composed of plant pathogens divided into 60 pathovars (Young 2010) belonging to nine genomospecies, as determined by DNA:DNA hybridisation (Gardan et al. 1999). On King’s B medium, the majority of these bacteria produce a fluorescent pigment visible under UV light (King et al. 1954). Bacteria that cause bacterial canker on stone fruit trees belong to three genomospecies (gs): gs 1—*P. syringae* pv. *syringae* (*Pss*); gs 2—*P. syringae* pv. *morsprunorum* race 1 (*Psm*1); and gs 3—*P. syringae* pv*. morsprunorum* race 2 (*Psm*2), *P. syringae* pv*. avii* (*Psa*) and *P. syringae* pv. *persicae* (*Psp*) (reviewed in Bultreys and Kałużna 2010). In Poland, three taxa were already described as present: *Pss*, *Psm*1 and *Psm*2. Recently, the new atypical taxon including bacteria that infect only cherries (mainly sour cherry) was also found (Kałużna data not published).

The diagnostics of bacterial canker are commonly based on isolation and phenotypic characterisation of the causal agent, including pathogenicity (Bultreys and Gheysen 1999; Vicente et al. 2004). The phenotypic tests LOPAT (Lelliott et al. 1966), GATTa and L-lactate utilisation (Lattore and Jones 1979) enable the determination of morphological, physiological and biochemical features of the bacteria. These features are used for identification of species and their discrimination into pathovars and races. However, this methodology requires the implementation of a high number of often laborious and time-consuming tests. Moreover, the obtained results can sometimes be ambiguous or difficult to interpret, and they are often not sufficient for proper strain classification (Vicente et al. 2004).

Concerning serological methods, the slide agglutination test, immunofluorescence and indirect-enzyme-linked immunosorbent assay (ELISA), with the antisera produced from live whole-cell antigens, were widely adopted for routine bacterial identification. However, nowadays these methods are less frequently used for the identification of bacteria that cause bacterial canker because of frequent cross-reactions with non-pathogenic bacteria. Furthermore, serological tests do not always provide a response in distinguishing isolates of *P. syringae* (Vicente et al. 2004).

Molecular methods are currently the most widely adapted and are considered very useful for the identification of bacterial canker causal agents and for studying their genetic diversity. For many years, the identification of the pathogen has been based on detection of genes encoding the toxins coronatine, syringomycin and the siderophore yersiniabactin (Bereswill et al. 1994; Sorensen et al. 1998; Bultreys and Gheysen 1999). However, it should be noted that the determination of presence of genes encoding for toxin production is not reliable for identification in itself and thus cannot be the only criterion for the classification of strains. In fact, strains of *Psm*1 and *Pss*, which do not have the ability to produce coronatine or syringomycin, respectively, are quite common (Ullrich et al. 1993; Renick et al. 2008; Kałużna et al. 2010a). On the other hand, although production of the siderophore yersiniabactin is now considered a stable feature of all *Psm*2 strains and could be a criterion for their identification, is should be mentioned that it is not an exclusive feature of strains of *Psm*2, since positive amplification with primers for the *irp1* gene (encoding this siderophore) was also confirmed in other pathovars of *P. syringae*, including the following: *antirrhini*, *apii*, *berberidis*, *delphinii*, *lachrymans*, *passiflorae*, *persicae*, *tomato*, *viburni*, *helianthi*, *tagetis* and *theae* (Bultreys et al. 2006).

In recent years, fingerprinting methods have been widely applied for the identification and genotyping of *P. syringae* through the analysis of repetitive regions (i.e. Enterobacterial Repetitive Intergenic Consensus (ERIC), BOX, Repetitive Extragenic Palindromic Elements (REP) and Insertion Sequence (IS50) sequences) (Ullrich et al. 1993; Weingart and Völksch 1997) and through PCR MP (Kałużna et al. 2010b). However, it should be taken into account that all fingerprinting methods require inclusion of the reference strains for comparison of obtained amplification patterns (Vicente and Roberts 2007; Gilbert et al. 2009), and, in the case of heterogeneous strains of *Pss* (Vicente et al. 2004; Renick et al. 2008; Kałużna et al. 2010a, b), it is difficult to determine affiliation of analysed strains to this taxon.

Despite the availability of different approaches for characterisation and genotyping of *P. syringae*, they require time-consuming and labour-intensive classical microbiological methods or complex analyses including comparison of amplification patterns and housekeeping gene sequencing. Therefore, there is still the need to develop a rapid and specific method of diagnosis that would allow the detection and identification of the causal agent of stone fruit bacterial canker (López et al. 2010). This specific, fast diagnostic system would be invaluable in the study on etiology of cankers on trunks and branches, which are similar to those caused by fungi of the genus *Leucostoma* (*Valsa*) and *Monilinia*, and also necrotic spots on leaves, which may be mistaken with those caused by *Prunus necrotic ring spot virus* or *Clasterosporium carpophilum*, especially late in the growing season. Moreover, the occurrence of gummosis on woody tissue often associated with bacterial infection may be related to the physiological response of the trees to damage caused by abiotic factors, such as frost, sunburn, periodic water flooding or mechanical damage, and is not due to biotic factors only (Saniewski et al. 2006).

Ideally, a novel diagnostic system would apply specific primers and the PCR technique, both conventional and real-time, making them more useful for a wide group of researchers according to available lab equipment, which allows for the detection and identification of the pathogen within a short amount of time. Additionally, such a system would undoubtedly be very useful in enforcing appropriate programmes to prevent and control disease occurrence in nurseries and orchards of stone fruit trees, especially sweet and sour cherry, where the damage is the most severe.

The aim of this study was to design and validate novel specific primers and to develop conventional and real-time PCR-based methodologies for rapid and specific detection of *Psm*1 and *Psm*2, with the aim of enhancing bacterial canker diagnostic procedures.

## Materials and methods

### Bacterial strains

Species and pathovar identification of previously uncharacterised *Pseudomonas* strains from our collection, obtained from stone fruit trees in Poland, was determined on the basis of phenotypic tests (i.e. Gram reaction with 3 % KOH (Suslow et al. 1982), LOPAT (Lelliott et al. 1966), GATTa and L-lactate utilisation (Lattore and Jones 1979). A total of 168 isolates were analysed. The reference strains *P. syringae* pv. *syringae*—LMG 1247, *P. syringae* pv. *morsprunorum* race 1—LMG 2222 and *P. syringae* pv. *morsprunorum* race 2—CFBP 3800 were included in all tests (Table 1). Additionally, type and not-type strains of other *P. syringae* pathovars (79) and related species (three) were included in the analysis (Table 2). The strains were kept at −75 °C in a mixture of glycerol (200 μl/ml) and phosphate-buffered saline (PBS) and streaked on King’s B medium (3.8 % Pseudomonas Agar F Difco, 1 % glycerol) (King et al. 1954) for routine culturing.

**Table 1:** Strains of *P. syringae* used in this study

Lp.	Strain number	Place (voivodeship/country) and year of isolation		Host-plant	Taxon based on LOPAT, GATTa/L
1.	58	Łódzkie, PL	2007	Sour cherry	Atypical taxon
2.	59	Łódzkie, PL	2007	Sour cherry	Atypical taxon
3.	61	Łódzkie, PL	2007	Sour cherry	Atypical taxon
4.	64	Łódzkie, PL	2007	Sour cherry	Atypical taxon
5.	65	Łódzkie, PL	2007	Sour cherry	Atypical taxon
6.	66	Łódzkie, PL	2007	Sour cherry	Atypical taxon
7.	69	Łódzkie, PL	2007	Sour cherry	Atypical taxon
8.	71	Łódzkie, PL	2007	Sour cherry	Atypical taxon
9.	72	Łódzkie, PL	2007	Sour cherry	Atypical taxon
10.	73	Łódzkie, PL	2007	Sour cherry	Atypical taxon
11.	74	Łódzkie, PL	2007	Sour cherry	Atypical taxon
12.	75	Łódzkie, PL	2007	Sour cherry	Atypical taxon
13.	76	Łódzkie, PL	2007	Sour cherry	Atypical taxon
14.	78	Łódzkie, PL	2007	Sour cherry	Atypical taxon
15.	80	Łódzkie, PL	2007	Sour cherry	Atypical taxon
16.	81	Łódzkie, PL	2007	Sour cherry	Atypical taxon
17.	82	Łódzkie, PL	2007	Sour cherry	Atypical taxon
18.	83	Łódzkie, PL	2007	Sour cherry	Atypical taxon
19.	86	Łódzkie, PL	2007	Sour cherry	Atypical taxon
20.	87	Łódzkie, PL	2007	Sour cherry	Atypical taxon
21.	88	Łódzkie, PL	2007	Sour cherry	Atypical taxon
22.	89	Łódzkie, PL	2007	Sour cherry	Atypical taxon
23.	90	Łódzkie, PL	2007	Sour cherry	Atypical taxon
24.	91	Łódzkie, PL	2007	Sour cherry	Atypical taxon
25.	93	Łódzkie, PL	2007	Sour cherry	Atypical taxon
26.	94	Łódzkie, PL	2007	Sour cherry	Atypical taxon
27.	95	Łódzkie, PL	2007	Sour cherry	Atypical taxon
28.	96	Łódzkie, PL	2007	Sour cherry	Atypical taxon
29.	118	Mazowieckie, PL	2007	Sour cherry	Atypical taxon
30.	119	Mazowieckie, PL	2007	Sour cherry	Atypical taxon
31.	120	Łódzkie, PL	2007	Sour cherry	Atypical taxon
32.	122	Łódzkie, PL	2007	Sour cherry	Atypical taxon
33.	211	Łódzkie, PL	2007	Sour cherry	Atypical taxon
34.	271	Silesian, PL	2007	Sour cherry	Atypical taxon
35.	374	Łódzkie, PL	2008	Sour cherry	Atypical taxon
36.	439	Łódzkie, PL	2008	Sour cherry	Atypical taxon
37.	909	Łódzkie, PL	2009	Sour cherry	Atypical taxon
38.	910	Łódzkie, PL	2009	Sour cherry	Atypical taxon
39.	949	Łódzkie, PL	2009	Sour cherry	Atypical taxon
40.	963	Lubelskie, PL	2009	Sweet cherry	Atypical taxon
41.	966	Lubelskie, PL	2009	Sour cherry	Atypical taxon
42.	967	Lubelskie, PL	2009	Sour cherry	Atypical taxon
43.	968	Lubelskie, PL	2009	Sour cherry	Atypical taxon
44.	969a	Lubelskie, PL	2009	Sour cherry	Atypical taxon
45.	969b	Lubelskie, PL	2009	Sour cherry	Atypical taxon
46.	970a	Lubelskie, PL	2009	Sour cherry	Atypical taxon
47.	970b	Lubelskie, PL	2009	Sour cherry	Atypical taxon
48.	971a	Lubelskie, PL	2009	Sour cherry	Atypical taxon
49.	971b	Lubelskie, PL	2009	Sour cherry	Atypical taxon
50.	972	Lubelskie, PL	2009	Sour cherry	Atypical taxon
51.	973	Lubelskie, PL	2009	Sour cherry	Atypical taxon
52.	981	Lubelskie, PL	2009	Sour cherry	Atypical taxon
53.	982	Lubelskie, PL	2009	Sour cherry	Atypical taxon
54.	1017	Łódzkie, PL	2009	Sour cherry	Atypical taxon
55.	1021	Łódzkie, PL	2009	Sour cherry	Atypical taxon
56.	791	No data	2001	Sour cherry	Atypical taxon
57.	441	Łódzkie, PL	2008	Plum	*Psm*1
58.	LMG 2222	No data, UK	1958	*Prunus avium*	*Psm*1
59.	25b	Łódzkie, PL	2007	Sweet cherry	*Psm*1
60.	28a	Łódzkie, PL	2007	Sweet cherry	*Psm*1
61.	29a	Łódzkie, PL	2007	Sweet cherry	*Psm*1
62.	38a	Łódzkie, PL	2007	Plum	*Psm*1
63.	98	Łódzkie, PL	2007	Sweet cherry	*Psm*1
64.	100	Łódzkie, PL	2007	Plum	*Psm*1
65.	107	Łódzkie, PL	2007	Plum	*Psm*1
66.	158	West Pomerania, PL	2007	Sweet cherry	*Psm*1
67.	174	West Pomerania, PL	2007	Sweet cherry	*Psm*1
68.	175	West Pomerania, PL	2007	Sweet cherry	*Psm*1
69.	177	West Pomerania, PL	2007	Peach	*Psm*1
70.	199	West Pomerania, PL	2007	Plum	*Psm*1
71.	201	West Pomerania, PL	2007	Plum	*Psm*1
72.	202	West Pomerania, PL	2007	Plum	*Psm*1
73.	203	West Pomerania, PL	2007	Plum	*Psm*1
74.	204	West Pomerania, PL	2007	Plum	*Psm*1
75.	205	West Pomerania, PL	2007	Plum	*Psm*1
76.	206	West Pomerania, PL	2007	plum	*Psm*1
77.	209	West Pomerania, PL	2007	Plum	*Psm*1
78.	213	Świętokrzyskie, PL	2007	Plum	*Psm*1
79.	214	Kuyavian-Pomeranian, PL	2007	Sweet cherry	*Psm*1
80.	215	Kuyavian-Pomeranian, PL	2007	Sweet cherry	*Psm*1
81.	216	Kuyavian-Pomeranian, PL	2007	Sweet cherry	*Psm*1
82.	217	Kuyavian-Pomeranian, PL	2007	Sweet cherry	*Psm*1
83.	218	Kuyavian-Pomeranian, PL	2007	Sweet cherry	*Psm*1
84.	219	Kuyavian-Pomeranian, PL	2007	Sweet cherry	*Psm*1
85.	220	Kuyavian-Pomeranian, PL	2007	Plum	*Psm*1
86.	221	Kuyavian-Pomeranian, PL	2007	Plum	*Psm*1
87.	250	Kuyavian-Pomeranian, PL	2007	Plum	*Psm*1
88.	274	Silesian, PL	2007	Plum	*Psm*1
89.	276	Silesian, PL	2007	Plum	*Psm*1
90.	280	Silesian, PL	2007	Plum	*Psm*1
91.	283	Silesian, PL	2007	Sweet cherry	*Psm*1
92.	291	Łódzkie, PL	2007	Sweet cherry	*Psm*1
93.	527	Mazowieckie, PL	2008	Sweet cherry	*Psm*1
94.	528	Mazowieckie, PL	2008	Sweet cherry	*Psm*1
95.	671	Lubelskie, PL	2008	Sweet cherry	*Psm*1
96.	1061	Łódzkie, PL	2009	Plum	*Psm*1
97.	701A	No data, PL	2005	Sweet cherry	*Psm*1
98.	702	No data, PL	1994	Plum	*Psm*1
99.	704	No data, PL	1994	Sweet cherry	*Psm*1
100.	710	Lower Silesian, PL	1996	Sweet cherry	*Psm*1
101.	755	No data, PL	1999	Plum	*Psm*1
102.	771	Łódzkie, PL	1999	Plum	*Psm*1
103.	782	No data, PL	2001	Sweet cherry	*Psm*1
104.	787	Mazowieckie, PL	2001	Plum	*Psm*1
105.	788	Łódzkie, PL	2001	Plum	*Psm*1
106.	793	Łódzkie, PL	2001	Plum	*Psm*1
107.	CFBP 3800	No data, UK	ND	*Prunus cerasus*	*Psm*2
108.	77	Łódzkie, PL	2007	Sour cherry	*Psm*2
109.	117	Mazowieckie, PL	2007	Sour cherry	*Psm*2
110.	266	Silesian, PL	2007	Sour cherry	*Psm*2
111.	417	Mazowieckie, PL	2008	Sour cherry	*Psm*2
112.	701	No data, PL	1994	Sour cherry	*Psm*2
113.	719	Łódzkie, PL	1997	Sour cherry	*Psm*2
114.	732	Łódzkie, PL	1997	Sour cherry	*Psm*2
115.	733	Łódzkie, PL	1997	Sour cherry	*Psm*2
116.	745	Łódzkie, PL	1999	Sour cherry	*Psm*2
117.	764	Mazowieckie, PL	1999	Sour cherry	*Psm*2
118.	LMG 1247	No data, UK	ND	*Syringa vulgaris*	*Pss*
119.	2905	No data/PL	1978	Sour cherry	*Pss*
120.	68	Łódzkie, PL	2007	Sour cherry	*Pss*
121.	103	Łódzkie, PL	2007	Sour cherry	*Pss*
122.	106	Łódzkie, PL	2007	Plum	*Pss*
123.	109	Łódzkie, PL	2007	Plum	*Pss*
124.	110	Łódzkie, PL	2007	Plum	*Pss*
125.	112	Łódzkie, PL	2007	Plum	*Pss*
126.	115	Łódzkie, PL	2007	Plum	*Pss*
127.	141	West Pomerania, PL	2007	Peach	*Pss*
128.	147	West Pomerania, PL	2007	Peach	*Pss*
129.	165	West Pomerania, PL	2007	Sweet cherry	*Pss*
130.	184	West Pomerania, PL	2007	Peach	*Pss*
131.	192	West Pomerania, PL	2007	Plum	*Pss*
132.	210	Łódzkie, PL	2007	Sour cherry	*Pss*
133.	222	Kuyavian-Pomeranian, PL	2007	Plum	*Pss*
134.	226	Kuyavian-Pomeranian, PL	2007	Plum	*Pss*
135.	227	Kuyavian-Pomeranian, PL	2007	Plum	*Pss*
136.	229	Kuyavian-Pomeranian, PL	2007	Plum	*Pss*
137.	233	Kuyavian-Pomeranian, PL	2007	Plum	*Pss*
138.	234	Kuyavian-Pomeranian, PL	2007	Plum	*Pss*
139.	235	Kuyavian-Pomeranian, PL	2007	Plum	*Pss*
140.	236	Kuyavian-Pomeranian, PL	2007	Plum	*Pss*
141.	237	Kuyavian-Pomeranian, PL	2007	Plum	*Pss*
142.	239	Kuyavian-Pomeranian, PL	2007	Plum	*Pss*
143.	240	Kuyavian-Pomeranian, PL	2007	Plum	*Pss*
144.	242	Kuyavian-Pomeranian, PL	2007	Plum	*Pss*
145.	244	Kuyavian-Pomeranian, PL	2007	Plum	*Pss*
146.	245	Kuyavian-Pomeranian, PL	2007	Plum	*Pss*
147.	247	Kuyavian-Pomeranian, PL	2007	Plum	*Pss*
148.	248	Kuyavian-Pomeranian, PL	2007	Plum	*Pss*
149.	256	Kuyavian-Pomeranian, PL	2007	Plum	*Pss*
150.	257	Kuyavian-Pomeranian, PL	2007	Sour cherry	*Pss*
151.	258	Kuyavian-Pomeranian, PL	2007	Sour cherry	*Pss*
152.	259	Łódzkie, PL	2007	Sweet cherry	*Pss*
153.	264	Łódzkie, PL	2007	Peach	*Pss*
154.	286	Silesian, PL	2007	Sweet cherry	*Pss*
155.	373	Łódzkie, PL	2008	Sour cherry	*Pss*
156.	376	Łódzkie, PL	2008	Sour cherry	*Pss*
157.	415	Świętokrzyskie, PL	2008	Plum	*Pss*
158.	420a	Mazowieckie, PL	2008	Sour cherry	*Pss*
159.	435	Mazowieckie, PL	2008	Sour cherry	*Pss*
160.	437	Łódzkie, PL	2008	Sour cherry	*Pss*
161.	442	Łódzkie, PL	2008	Plum	*Pss*
162.	460	Podkarpackie, PL	2008	Sour cherry	*Pss*
163.	663	Lubelskie, PL	2008	Sour cherry	*Pss*
164.	914	Kuyavian-Pomeranian, PL	2009	Sour cherry	*Pss*
165.	959	Lubelskie, PL	2009	Sour cherry	*Pss*
166.	702A	Łódzkie, PL	2005	Plum	*Pss*
167.	753	Łódzkie, PL	1999	Apricot	*Pss*
168.	757	Mazowieckie, PL	1999	Plum	*Pss*
169.	760	Mazowieckie, PL	1999	Sour cherry	*Pss*
170.	762	No data, PL	1999	Apricot	*Pss*
171.	763	No data, PL	1999	Sour cherry	*Pss*

*LOPAT*—levan production from sucrose (*L*), presence of oxidase (*O*), ability to cause rot on potato tubers (*P*, pectolytic activity), presence of arginine dihydrolase (*A*), hypersensitive reaction (HR) on tobacco plants; *GATTA*—gelatine hydrolysis (*G*), aesculin hydrolysis (*A*, activity of the β-glucosidase), tyrosinase activity (*T*), utilisation of tartrate (*Ta*); test of L-lactate utilisation (*L*); *PL* Poland, *UK* United Kingdom

**Table 2:**
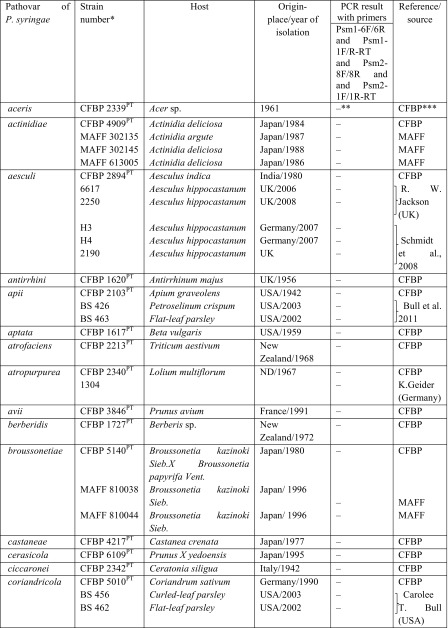

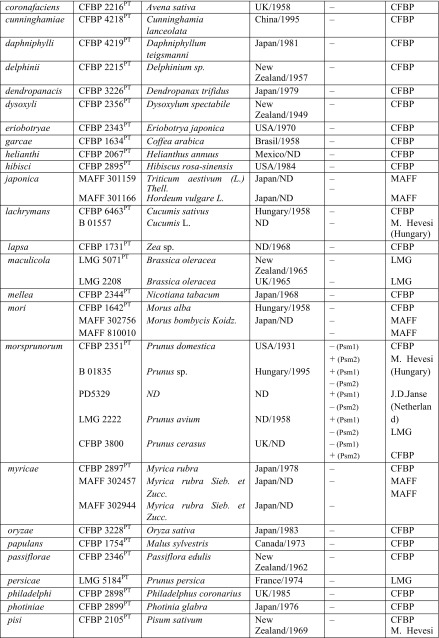

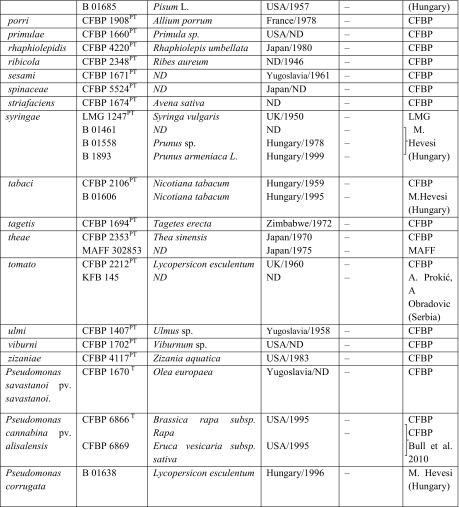
Results of specificity of designed primers in reactions with DNA of different pathovars of *Pseudomonas syringe* and other *Pseudomonas* species tested

### DNA isolation

Bacterial DNA was isolated using the method described by Aljanabi and Martinez (1997), with slight modifications described by Kałużna et al. (2012). DNA was diluted to a final concentration of 10 ng/μl and kept at −20 °C for further analysis.

### PCR melting profile

**Fig. 1:**
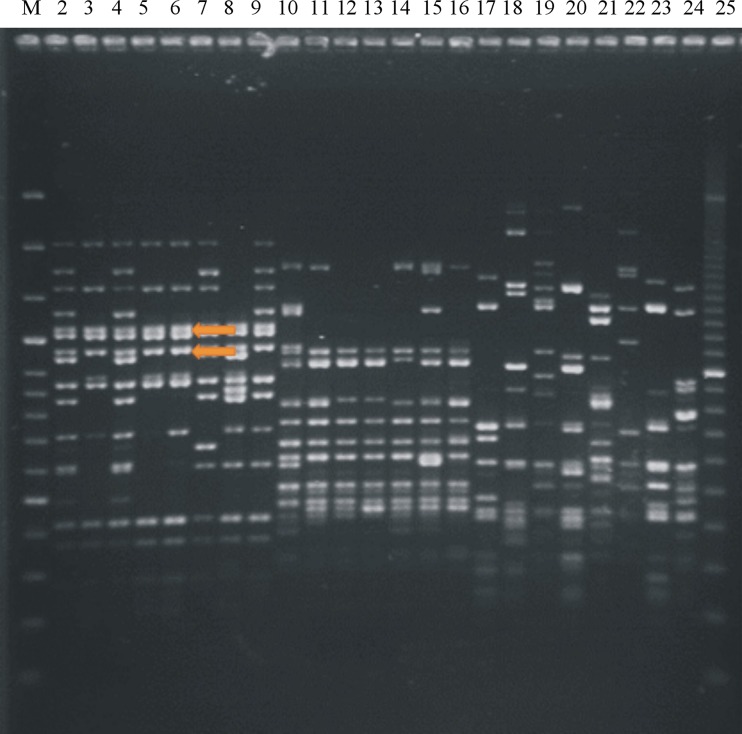
Electrophoretic patterns obtained after polymerase chain reaction melting profile (PCR MP) of fluorescent Pseudomonads with primer Pst1: Lane 1—*M*—marker 100-bp ladder (Genoplast, Rokocin, Poland); pathovar *morsprunorum* race 1 isolates: *2*—LMG 2222, *3*—702, *4*—710, *5*—755, *6*—787, *7*—782, *8*—793, *9*—701A; pv. *morsprunorum* race 2 isolates: *10*—CFBP 3800, *11*—719, *12*—733, *13*—732, *14*—745, *15*—764, *16*—701; pv. *syringae* isolates: *17*—LMG 1247, *18*—2905, *19*—760; *20*—762, *21*—702A, *22*—757, *23*—753, *24*—763, *25*—M—marker 100-bp PCR Molecular Ruler (Bio-Rad, Hercules, USA)

**Fig. 2:**
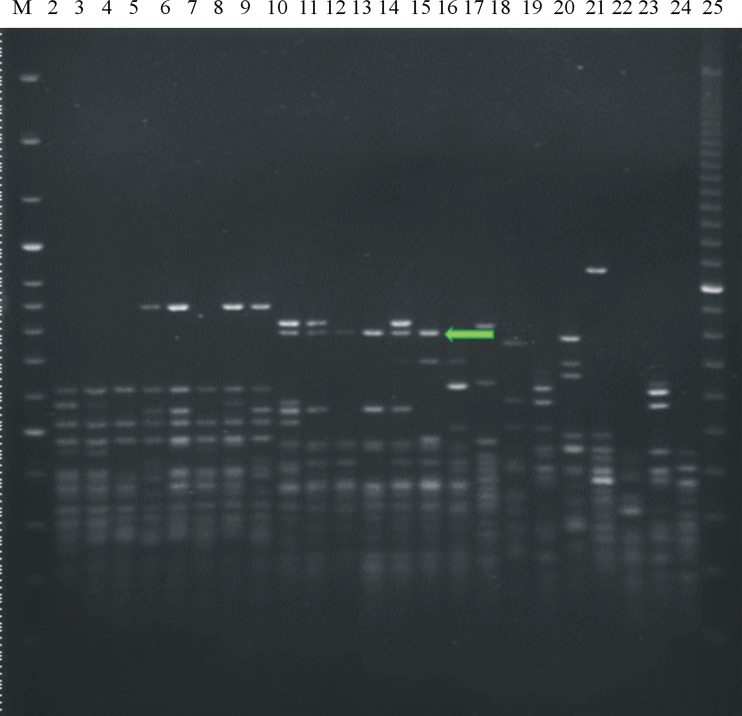
Electrophoretic patterns obtained after polymerase chain reaction melting profile (PCR MP) of fluorescent Pseudomonads with primer Taq1: Lane 1—*M*—marker 100-bp ladder (Genoplast, Rokocin, Poland); pathovar *morsprunorum* race 1 isolates: *2*—LMG 2222, *3*—25b, *4*—28a , *5*—107, *6*—201, *7*—701A, *8*—755, *9*—771; pv. *morsprunorum* race 2 isolates: *10*—CFBP 3800, *11*—77, *12*—701, *13*—732, *14*—733, *15*—745, *16*—764; pv. *syringae* isolates: *17*—LMG 1247, *18*—2905, *19*—68; *20*—110, *21*—141, *22*—286, *23*—415, *24*—763, *25*—M—marker 100-bp PCR Molecular Ruler (Bio-Rad, Hercules, USA)

A slightly modified method of PCR MP described by Masny and Płucienniczak (2003) was used. An amount of 100 ng of DNA from 23 *Pseudomonas* strains (Figs. 1 and 2) was digested with *Pst*I endonuclease (10 U/μl; Promega Corporation, Madison, WI, USA) or *Taq*I (10 U/μl; Thermo Scientific, Vilnius, Lithuania) according to the manufacturer’s instructions. Digested DNA was ligated with two oligonucleotides forming an adaptor: DNA digested by *Pst*I endonuclease with a PstI adaptor—5′-TGTACGCAGTCTAC-3′/5′-CTCGTAGACTGCGTACATGCA-3′ (Waugh et al. 1997) and DNA digested by *Taq*I endonuclease with a TaqI adaptor—5′-GACGATGAGTCCTGAC-3′/5′-CGGTCAGGACTCAT-3′ (Ajmone-Marsan et al. 1997). PCR amplification was performed separately for *Pst*I- or *Taq*I-digested DNA in a 25-μl reaction mixture containing the following: 1 μl of ligation mixture; 0.4 U of GoTaq DNA polymerase (Promega, Madison, WI, USA) for *Pst*I and 0.4 U of Dream Taq Green DNA Polymerase (Thermo Scientific, Vilnius, Lithuania) for *Taq*I; and 1× of appropriate Taq polymerase buffer, 0.2 mM of dNTPs and 1 μM of each primer (PstI-0—5′-GACTGCGTACATGCAG-3′ for *Pst*I-digested DNA (Waugh et al. 1997) or TaqI-0—5′-GACGATGAGTCCTGACCGA-3′ for *Taq*I-digested DNA (Ajmone-Marsan et al. 1997)). The amplification reactions were conducted in a Biometra T3000 thermocycler (Biometra, Göttingen, Germany) with the following conditions: initial step of 72 °C for 5 min; 30 cycles at 86.5 °C for *Pst*I and 83 °C for *Taq*I for 40 s, 55 °C for 40 s and extension at 72 °C for 90 s; and final extension at 72 °C for 10 min. PCR products from each reaction and the O’GeneRuler 100-bp DNA Ladder Plus (Thermo Scientific, Vilnius, Lithuania) were separated on a 1.5 % agarose gel in 0.5× TBE buffer (0.045 M tris-boric acid, 0.001 M EDTA, pH 8.0) and electrophoresis was ran at 5–7 V/cm of gel. After staining with an ethidium bromide solution (0.5 μg/ml), the obtained amplification profiles were visualised under UV light. The same conditions were used in all subsequent electrophoresis.

### Selection of specific fragments

Based on the results of genetic analyses using PCR MP, DNA fragments characteristic of *Psm*1 and *Psm*2 strains were selected. The fragments were excised from the gel, purified with the DNA AxyPrep Gel Extraction Kit (Axygen Scientific, Inc. Union City, CA, USA) and cloned into the pGEM T-Easy vector (Promega, Madison, WI, USA) according to the manufacturer’s instructions. The resulting ligation mixture was used to transform *Escherichia coli* JM109 competent cells (Promega, Madison, WI, USA). The cloned fragments were sequenced with universal primers M13Rev 5′-CAGGAAACAGCTATGAC-3′ and M13 (−40) 5′-GTTTTCCCAGTCACGAC-3′ at Genomed S.A. (Warsaw, Poland). The sequences obtained were assembled using the SeqMan software package LASERGENE (DNASTAR, Madison, USA).

### Design of SCAR primers

The sequences of specific fragments for *Psm*1 and *Psm*2 were used to design the SCAR primers, for both conventional and real-time PCR, with the PrimerSelect programme of the LASERGENE package (DNASTAR). Different primer pairs were designed for conventional PCR (five for *Psm*1 and 7 for *Psm*2) and real time PCR (four for each taxa). All primer sequences and their potential amplification reaction products were checked for homology (June 2015) to other sequences deposited in the GenBank database using the ‘blastn’ algorithm (Altschul et al. 1997). Selected primers were synthesised at Genomed S.A.

### Dot blot hybridisation

High-throughput specificity assays were carried out using a dot blot platform, essentially as previously described (Albuquerque et al. 2011). PCR amplicons obtained using primers Psm1-6F/6R, with template DNA from strain *Psm* 28a (race 1), and primers Psm2-8F/8R, with *Psm* 77 (race 2), were purified using the GFX PCR and Gel Band Purification Kit (GE Healthcare, Buckinghamshire, UK) and labelled with digoxigenin, using the DIG-High Prime DNA labelling kit (Roche, Basel, Switzerland) in order to obtain the two tested hybridisation probes Psm1 and Psm2, respectively.

Amounts of 100 ng of heat-denatured DNA from each bacterial strain were transferred to a nylon membrane using a Bio-Dot apparatus (Bio-Rad, Hercules, USA). Hybridisation was carried out overnight at 68 °C with a final probe concentration of 100 ng/mL, and the washing and detection steps were carried out according to the DIG application manual (Roche). The chemiluminescent signal indicative of probe–target hybrids was detected using a Molecular Imager ChemiDoc XRS+ System (Bio-Rad), with all pixels below saturation point.

### Conventional and real-time PCR amplifications

Amplification reactions with the two selected primer pairs, one specific for the strains of *Psm*1 and the second specific for *Psm*2, were performed in a Biometra T3000 thermocycler (Biometra, Göttingen, Germany). The reaction mixture in 15 μl of total reaction volume contained 10 ng of DNA, 0.4 U of Dream DNA Polymerase (Promega, Madison, WI, USA), 1× reaction Dream Taq Green buffer (Thermo Scientific, Vilnius, Lithuania), 0.15 mM dNTPs and 0.7 mM of each primer. The following experimentally determined amplification conditions were used: initial denaturation at 94 °C for 4 min; 30 cycles at 94 °C for 45 s, 55–62 °C for 45 s for primers Psm1-6F and Psm1-6R (for detection of *Psm*1 strains) and 50–58 °C for 45 s for primers Psm2-8F and Psm2-8R (for detection of *Psm*2 strains) and 72 °C for 1 min; and final extension at 72 °C for 10 min. The resulting PCR products were separated by electrophoresis on 1.5 % agarose gels as described above.

Real-time PCR with SYBR Green I was conducted in the Bio-Rad CFX96 with SsoAdvanced SYBR Green Supermix (Bio-Rad, Hercules, USA). The reaction mixture in 20 μl of total volume contained 1× reaction SYBR Green Supermix and 0.5 mM of each of the following primers: Psm1-1F-RT/Psm1-1R-RT for *Psm*1 and Psm2-1F-RT/Psm2-1R-RT for *Psm*2. Bacterial DNA was used as a template (10 ng per PCR reaction). No-template reactions were used as negative controls. The PCR programme was started from one cycle of denaturation at 98 °C for 130 s, followed by 35 cycles at 95 °C for 10 s and then 60 °C for 15 s, finished by a melting curve analysis for verification of the specificity of amplification in real-time PCR products. Progressive denaturation of products was carried out at a rising temperature, starting from 65 °C and continuing to 95 °C, with 0.5 °C of increment for 5 s each.

### Specificity of designed primers and their usefulness in detection in plant material

In the first stage of this part of the study, the specificity of the two designed primer pairs was determined with PCR using DNA from all strains of *Psm*1, *Psm*2 and *Pss* as well as strains of atypical taxa (Table 1). In the second stage, the primers were tested with DNA from other *P. syringae* pathovars and related species (Table 2).

In order to assess the suitability of the designed primers for the detection of *Psm*1 and *Psm*2 strains in plant material, several leaves, shoots and fruits of sweet cherry, sour cherry and plum were collected. Amounts of 100 mg of crushed/cut plant tissue of each organ were placed in 1.9 ml of PBS buffer. For each type of tissue (organ) and host plant, two tubes were prepared (18 tubes in total). One hundred microlitres of bacterial suspension (10^5^ cfu/ml) of the *Psm*1 reference strain (LMG 2222) or the *Psm*2 reference strain (CFBP 3800) were added to nine of the samples (one of each organ and of each plant). One hundred microlitres of sterile water were added to the remaining nine samples, which were tested to verify the purity of the plant material. After 1 h of shaking incubation at 26 °C, 1 ml of washing liquid separate from each of all 18 samples was centrifuged; the resulting pellet was suspended in 100 μl of TE buffer, and the DNA was isolated using a Genomic Mini DNA Extraction Kit (A&A Biotechnology, Gdynia, Poland) according to the manufacturer’s instructions.

### PCR limit of detection

The limit of detection of PCR using the SCAR primers was evaluated using DNA extracted from pure bacterial cultures, DNA extracted from plant material that was mixed with suspensions of bacteria and bacterial genomic DNA (gDNA). A PCR assay was carried out with decimal dilutions of bacterial suspensions of strain LMG 2222 or CFBP 3800 (from ~10^8^ to 10^0^ cfu/ml). DNA was isolated from 1 mL of each dilution using a Genomic Mini DNA Extraction Kit (A&A Biotechnology) according to the protocol supplied by the manufacturer. To determine the limit of detection of bacteria in the plant material, 100-mg portions of stems and leaves of sweet cherry (for *Psm*1 primers) or sour cherry (for *Psm*2 primers) and 100 μl of the previously prepared 10-fold serial dilutions of bacterial suspensions (from ~10^8^ to 10^0^ cfu/ml) or 100 μl of sterile water, used as a control of material purity, were added to 1.9 mL of PBS buffer and shaken for 30 min at 26 °C. After incubation, the washings were centrifuged (8,000 rpm, 5 min); the resulting pellet was suspended in 100 μl of TE buffer, and DNA was isolated using the Genomic Mini DNA Extraction Kit (A&A Biotechnology) according to the manufacturer’s instructions. The sensitivity of gDNA detection was checked using 2-fold serial dilutions of gDNA isolated (11 ng to ~11 fg per PCR reaction for *Psm*1 and 14 ng to ~14 fg per PCR reaction for *Psm*2) using the method described by Aljanabi and Martinez (1997), with slight modifications described by Kałużna et al. (2012). The PCR efficiency was calculated from the slope of the standard curve generated for each run in the following equation *E* = 10^(−1/slope)^ where *E* = 2 and corresponds to 100 % efficiency (Ramakers et al. 2003).

## Results

### Phenotypic characterisation

All 168 isolates have been classified into species *P. syringae* LOPAT group Ia. GATTa and L-lactate utilisation tests allowed further discrimination of pathovars and races: 49 isolates were identified as *P. syringae* pv. *morsprunorum* race 1 (*Psm*1), 10 as race 2 of this pathovar, 53 as pathovar *syringae* (*Pss*) and 56 as belonging to atypical taxa, having most of the features of *Pss* without, however, the ability of esculine hydrolysis (lack of β-glucosidase activity) (Table 1).

### PCR MP

To select specific fragments of the taxon, the PCR MP method was applied using DNA from different strains of *P. syringae* (Figs. 1 and 2; Table 1). The obtained PCR MP patterns corresponded to phenotypically determined pathovars and races. Similar electrophoretic patterns were obtained for races within pathovar *morsprunorum*, confirming their homogeneity; however, different patterns were observed for strains belonging to pathovar *syringae*. For *Psm*1 and *Psm*2, the products that were specific and were shared between all strains of each taxa were selected, cloned and sequenced. Two products specific for *Psm*1 (after digestion by *Pst*I) had sizes of 1,208 and 1,128 bp, while the unique amplification product (after digestion by *Taq*I) for strains of *Psm*2 was 781 bp long. No specific and unique band was found for strains of *Pss*.

### Design of SCAR primers

The nucleotide sequences obtained for the *Psm*1 and *Psm*2 fragments were used to design different SCAR primers. After validation, the most specific primers for conventional and real-time PCR were selected (Table 3). A BLAST analysis of selected primer sequences showed no similarity to any bacterial sequences in GenBank.

**Table 3:** Primers specific for strains of *Psm*1 and *Psm*2

	Primer name	Primer sequence	T_m_	Product length
Conventional PCR	Psm1-6F	5′-TGTTCCCGGCCATCCAATA-3′	51.1 °C	793 bp
	Psm1-6R	5′-ATCCGCATCAGTCAAAATAGTCAT-3′	52.3 °C	
	Psm2-8F	5′-CTTTTTAGATGGTGAGGTTTTGTA-3′	50.6 °C	410 bp
	Psm2-8R	5′-ACTTTCGGATCATCGTTTTCTA-3′	49.2 °C	
Real-time PCR	Psm1-1F-RT	5′-TCCCGGCCATCCAATACTTTACG-3′	57.1 °C	101 bp
	Psm1-1R-RT	5′-ACGCTTCATGGTGTCTTGTTTA-3′	51.1 °C	
	Psm2-1F-RT	5′-GGTTTGCCTTTTCCTCAG-3′	48 °C	104 bp
	Psm2-1R-RT	5′-ATTGCATTACTTCTTTGTTGC-3′	46.5 °C	

*F* forward primer, *R* reverse primer, *RT* real-time, *Tm* melting temperature

### Dot blot hybridisation

**Fig. 3:**
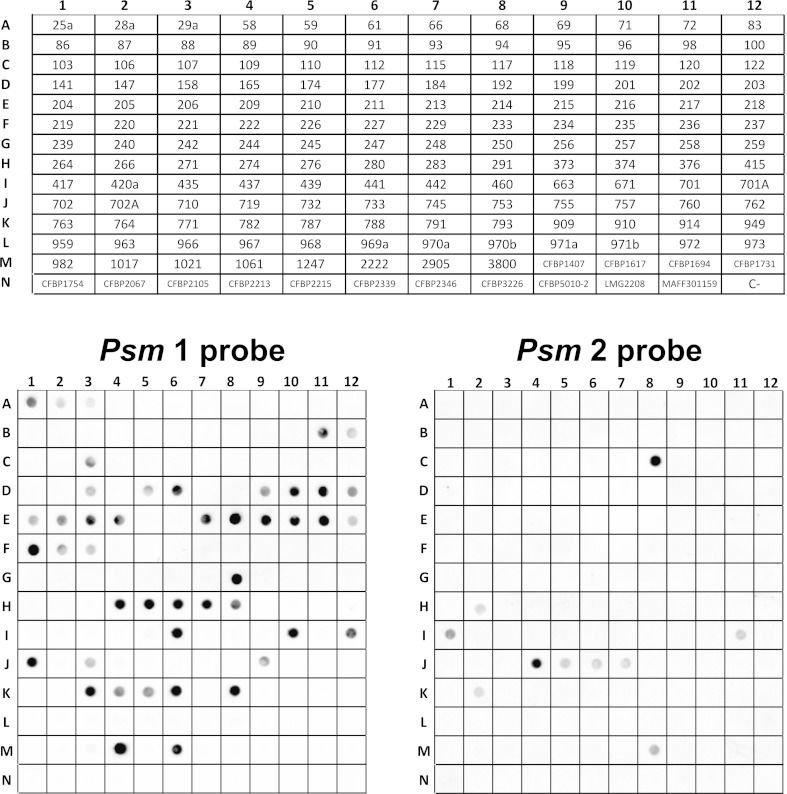
Dot blot validation of probes Psm1 and Psm2. The probes were evaluated with total DNA from 167 strains; including *P. syringae* strains isolated from stone fruit trees, reference strains and others pathovars of *P. syringae* from the CFBP culture collection. The *table grid* above represents the coordinates of each strain tested in the dot blot, which are identified by their abbreviations further detailed in Tables 1 and 2. Positive hybridization signals are visualised as *dark dots*

The dot blot results confirmed the high specificity of the selected markers towards the target pathogens. Using probe Psm1, positive hybridisation results (dark dots) were observed with all tested Psm1 strains, and no unspecific hybridisation was observed with DNA from any non-*Psm*1 pseudomonads. Similarly, probe *Psm*2 was exclusively specific for the tested *Psm*2 strains. Additionally, the hybridisation results showed that the selected DNA regions were present in all their respective target strains, confirming their stability (Fig. 3).

### Specificity of designed primers and usefulness in detection in plant material

**Fig. 4:**
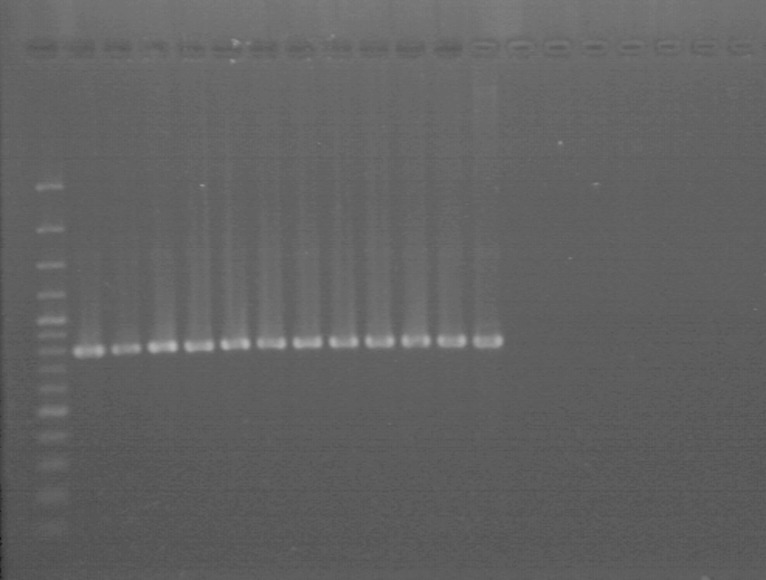
Evaluation of primers Psm1-6F and Psm1-6R for identification of *P. syringae* pv. *morsprunorum* race 1: M—O’GeneRuler 100–3000 bp (Thermo Scientific, Vilnius, Lithuania), strains *Psm*1: strains *Psm*1: 2—LMG 2222, 3—28a, 4—29a, 5—38a, 6—175, 7—199, 8—201, 9—203, 10—205, 11—274, 12—755, strains *Psm*2:13—CFBP 3800, 14—732, strains *Pss*: 15—LMG 1247, 16—760, strains of atypical taxon: 17—58, 18—970a, 19—K-

**Fig 5:**
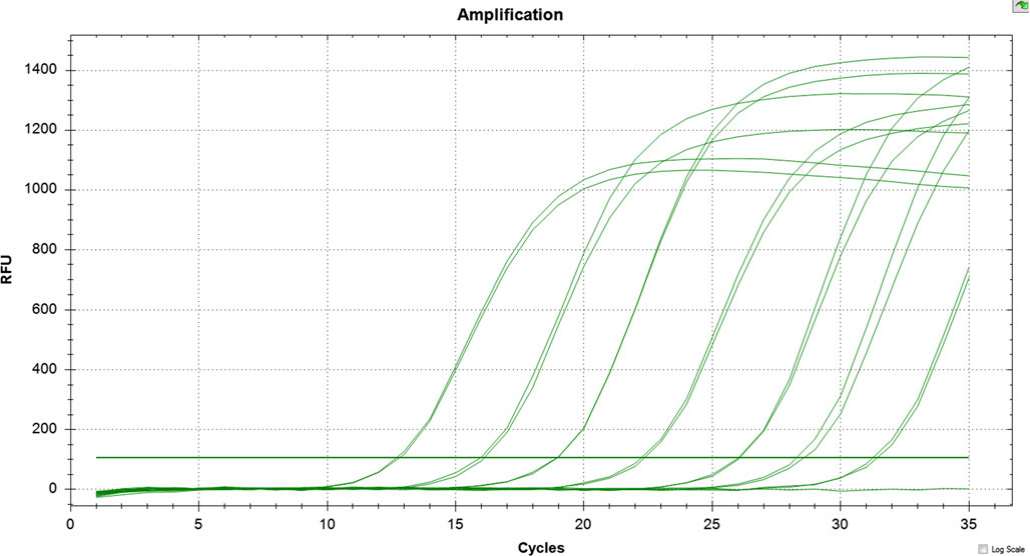
Real-time PCR with SYBR Green I (Bio-Rad, Hercules, USA) for specific detection of DNA from suspension of *Psm*1 strain LMG2222 (example). Fluorescence signal is related to the amount of template. Samples from 10^6^, 10^5^, 10^4^, 10^3^, 10^2^, 10^1^ and 10^0^ cfu/reaction

**Fig. 6:**
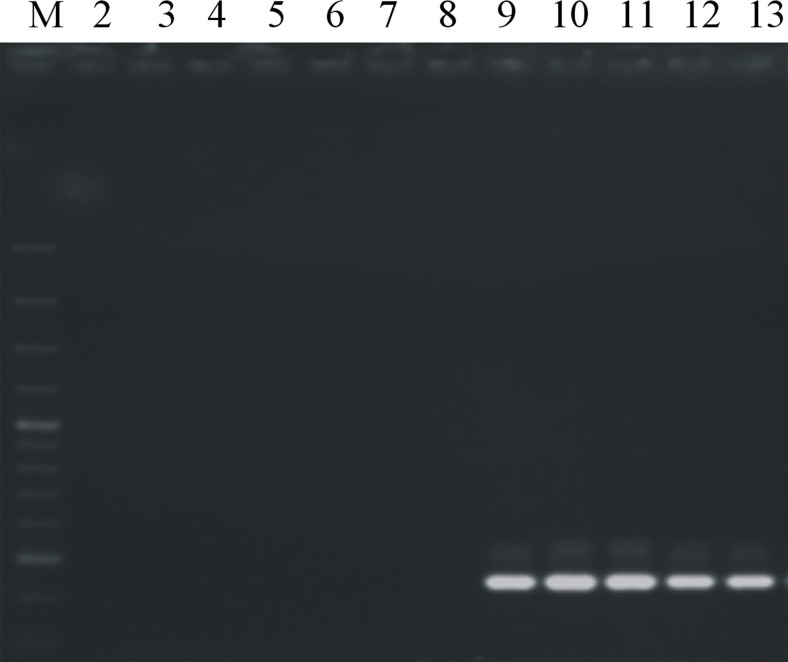
Evaluation of primers Psm2-8F and Psm2-8R for identification of *P. syringae* pv. *morsprunorum* race 2: *M*—O’GeneRuler 100–3000 bp (Thermo Scientific, Vilnius, Lithuania), strains *Psm*1: *2*—25b, *3*—250, *4*—788; strains *Pss*: *5*—68, *6*—110; strains of atypical taxon: *7*—61, *8*—970a; strains *Psm*2: *9*—77, *10*—CFBP3800, *11*—77, *12*—745, *13*—764

The PCR assays using DNA from all tested *P. syringae* strains including reference strains (Table 1), as well as DNA from strains of other species within the *Pseudomonas* genus (Table 2), showed that all the designed primers were specific for their respective taxa. PCR assays using primers Psm1-6F/6R and Psm1-1F-RT/1R-RT, specific for *Psm*1, successfully amplified the expected PCR products 793 bp (Fig. 4) and 101 bp (Fig. 5), respectively, using DNA from all strains of *Psm*1. No amplification was observed when DNA from strains identified as *Psm*2 or *Pss* and strains of atypical taxa were used. Amplification using primers Psm2-8F/8R and Psm2-1F-RT/1R-RT, designed for detection of *Psm*2, was achieved with DNA from all strains of *Psm*2, resulting in PCR products of expected lengths of 410 bp (Fig. 6) and 104 bp, respectively. No increase in fluorescence was observed with DNA from *Psm*1 or *Pss* and strains of atypical taxa. The melting curves of the reaction products obtained from real-time PCR revealed a single peak with a melting temperature of 80 °C or 77 °C for *Psm*1 and *Psm*2, respectively. Also, neither unexpected nor additional peaks in the product melting curves were observed, which clearly excluded possibilities or tendency of the primers to form dimers. Moreover, none of the four tested primer pairs amplified the DNA of 79 strains of other pathovars of *P. syringae* and other species (Table 2).

The usefulness of the designed primers for detection of *Psm*1 and *Psm*2 strains in plant material was assessed with PCR assays using DNA extracted from a mixture of plant tissues and a suspension of target bacteria. The results confirmed the specificity of selected primer-pairs since positive amplification was achieved in mingled samples, while no nonspecific amplification was observed in samples without bacteria addition. Additionally, these assays showed that the proposed PCR detection methodology was not affected by potential inhibitors present in plant samples.

### Limit of detection of *P. syringae* pv. *morsprunorum* for conventional and real-time PCR

Both tested primer pairs designed for conventional PCR allowed for the detection of 10^0^ cfu/reaction of *Psm*1 and 10^1^ *Psm*2 in pure culture. Regarding the presence of bacteria in different organs of sweet and sour cherries, it was possible to detect 10^0^ and 10^1^ cfu/reaction for sweet cherry leaves and shoots, respectively, using the *Psm*1-specific primers and 10^2^ cfu/reaction for sour cherry leaves and shoots using the *Psm*2-specific primers. The sensitivity (LOD, limit of detection) of the detection in the conventional PCR assay was ~4 pg for *Psm*1 strain 199 and ~5 pg for *Psm*2 strain 745 when aliquots of serial 2-fold dilutions of purified DNA were used which corresponds to the order of magnitude ~10^1^–10^2^ cfu/reaction.

Both tested primer pairs designed for *Psm*1 and *Psm*2 strains using real-time PCR allowed the detection of 10^0^ cfu/reaction of *Psm*1 or *Psm*2 in pure culture and in plant material. Only the expected products and a single peak with melting temperature were obtained. Standard curves using template DNA from bacterial suspensions, DNA from plant material with additions of bacterial suspensions and bacterial gDNA showed high amplification efficiency and linearity of the data (Table 4). An exception occurred for the products obtained from shoots of sweet cherry with additions of bacterial suspensions of *Psm*1. Although linearity was quite good, the noted efficiency of 83 % was not in the range considered acceptable (90–110 %). Moreover, the efficiency obtained for the mixture of shoots of sour cherry and *Psm*2 suspension when testing with primers for *Psm*2 was also lower compared to DNA template from sour cherry leaves and bacterial suspension alone. The sensitivity (LOD) of the detection in the real-time PCR assay when using gDNA ranged from ~30 to 100 fg for *Psm*1 strain 199 and ~10 to 50 fg for *Psm*2 strain 745 when 1.0-μl aliquots of serial 2-fold dilutions of purified DNA were used which corresponds to the order of magnitude ~10^0^ cfu/reaction (Table 4).

**Table 4:** Important parameters of real-time polymerase chain reaction (PCR) runs evaluated through the analysis of standard curves generated with different DNA templates of *P. syringae* pv. *morsprunorum* races 1 and 2

Template	E (%)^a^	R2 ^b^	Slope ^c^	*Y* = int ^d^
*Psm*1 (DNA from bacterial suspension)	103	0.998	−3.252	35.445
*Psm*1+sweet cherry leaves	99.7	0.965	−3.328	36.551
*Psm*1+sweet cherry shoots	83.0	0.989	−3.810	43.932
*Psm*1 gDNA	99.2	0.997	−3.342	18.425
*Psm*2 (DNA from bacterial suspension)	99.8	0.995	−3.326	33.093
*Psm*2+sour cherry leaves	99.3	0.999	−3.338	32.451
*Psm*2+sour cherry shoots	91.4	0.994	−3.548	35.130
*Psm*2 g DNA	99.2	0.991	−3.342	17.805

^a^ *E* = PCR efficiency; ideally the efficiency should be 100 %, meaning that for each cycle the amount of product doubles; high/acceptable amplification efficiency (90–110 %). Efficiency = 10^(−1/slope)^ − 1

^b^R2 is a measure of data linearity amongst technical replicates of serial dilutions; indicates how good one value is in predicting another; R2 = 1 is perfect

^c^The slope of the log-linear phase of the amplification reaction is a measure of reaction efficiency. To obtain accurate and reproducible results, reactions should have an efficiency as close to 100 % as possible, equivalent to a slope of −3.32

^d^ *Y* = int represents the value of Ct where the curve crosses the y-axis

## Discussion

In this study, the methods and tools enabling the rapid and highly specific identification and detection of bacterial canker causal agent *P. syringae* pv. *morsprunorum* races 1 and 2 were developed. The methods based on the use of specific primers designed for conventional and real-time PCR allow in routine testing for omitting the application of often time-consuming methods of classical microbiology, fingerprinting methods or housekeeping gene sequence analysis used until now by other authors (Vicente and Roberts 2007; Gilbert et al. 2009). Of course in critical cases (i.e. first reports, claims, etc.) these other methods are still indispensable. Our newly developed methods and tools are very useful and invaluable in both epidemiological studies and in development of protection programmes for stone fruits against bacterial canker.

Using the genetic fingerprinting PCR MP method, we demonstrated the diversity of *P. syringae* strains, which was very important in the selection of specific DNA fragments for two races of *P. syringae* pv. *morsprunorum*. Based on the obtained nucleotide sequences of these fragments, *Psm*1- and *Psm*2-specific SCAR primers were designed. The specificity of the designed primers for *Psm* and amplified regions was confirmed by BLAST, since the fragments did not show (at present) any significant similarity hits within the NCBI database. Due to the high electrophoretic profile heterogeneity obtained for *Pss* strains arising from their high genetic diversity confirmed already by other authors (Vicente and Roberts 2007; Gilbert et al. 2009; Kałużna et al. 2010a, b), it was not possible to find a common DNA fragment for all strains belonging to this taxon.

Commonly used methods for designing SCAR primers include rep-PCR (repetitive PCR) (Sangdee et al. 2013), randomly amplified polymorphic DNA (RAPD) (Liu et al. 2012; Cheng et al. 2015), amplified fragment length polymorphism (AFLP) (Zhang et al. 2012), PCR with universal rice primers (URP-PCR) (Lim et al. 2009) and inter-simple sequence repeat (ISSR) (Giaj Merlera et al. 2015). Although the PCR MP method was described so far as helpful in the study of genetic diversity of bacteria and yeast (Leibner-Ciszak et al. 2010; Kałużna et al. 2010b, 2014; Zasada et al. 2014), it has not been previously reported to be used for the selection of SCAR markers. In this work, the PCR MP is for the first time used for the design of SCAR primers specific for detection of plant pathogenic bacteria.

The results obtained in this study showed that the designed SCAR primers can be applied for specific, direct detection of strains belonging to *Psm*1 or *Psm*2, both in pure culture and infected plant material. Their specificity was confirmed by PCR, using DNA from several *Pseudomonas* spp. strains, which showed that positive amplification occurred only with DNA of the targeted taxa strains. This is especially significant in the case of strains of atypical taxa and pathovars of *P. syringae* (i.e. pv. *syringae* and pv. *avii*, which also infect cherry (Ménard et al. 2003; Renick et al. 2008)) to exclude that symptoms are connected to another taxa/pathogen or to abiotic factors. Importantly, when testing the developed primers in conventional PCR, using DNA isolated from a mixture of plant material and bacteria of *Psm*1 or *Psm*2, the suppression of amplification by potential plant inhibitors like polyphenols and pesticide residues, as reported by Puławska et al. (1997), was not found. Additionally, for DNA from the asymptomatic plant material without addition of bacterial DNA, no positive amplification was observed. This means that the designed primers did not react with DNA of potential bacteria naturally inhabiting the plant material, which is essential to prevent false-positive diagnostic results. However, in the case of real-time PCR, which is the more sensitive method, some effects of plant material were noted. Although standard curves using different template DNA showed the high amplification efficiency and linearity of the data for the majority of DNA tested, for shoots of sweet cherry with additions of bacterial suspensions the efficiency was below the range considered acceptable, indicating higher dilution of those templates than expected. Also, a decrease of efficiency (Table 4) in the case of sour cherry shoots was observed. The results therefore may indicate the influence of shoots for more sensitive real-time PCR reactions.

The designing of primers for both systems, conventional and real-time PCR, makes the developed diagnosis system more accessible to a wider group of researchers, as many laboratories do not have access to special equipment or specialised personnel to perform the real-time PCR or have less funds. However, as described, the real-time PCR procedure is much faster (whole reaction with melting curve analysis is about 1 h from the beginning with SsoAdvanced SYBR Green Supermix); it allows the use of DNA quickly extracted from pure culture by the boiling method, without loss of detection resolution, and also excludes additional time-consuming post-PCR processes (i.e. agarose gel electrophoresis). Therefore, using this technique, it is possible to obtain a very fast response about the causal agent of the disease. However, it should be noted that this system is highly sensitive and that false-positive results can occur. The risk of false-positive results due to cross-contamination during preparation of the PCR can be minimised by using negative controls and high discipline during work (e.g., application of tips with filters during the DNA isolation step). Additionally, positive results obtained during those of the final PCR cycles should be treated as suspect only, for which additional, more detailed investigations should be conducted. Moreover, during all the assays the melting curve analysis is recommended to exclude nonspecific amplicons (as a consequence of which are visible in each run as the rest of the analysed specific ones). Dot blot hybridisation confirmed that the two selected DNA regions were highly specific for their target genomospecies and stable amongst all tested isolates of either *Psm*1 or *Psm*2, which is essential for preventing false-positive and false-negative results, respectively as much as possible.

In summary, when compared with so-far available methods for identification and differentiation of causal agents of stone fruit bacterial canker based on phenotypic characters, fingerprinting methods or MLST, the use of pathovar-specific primers allowed for greatly shortening the time required for diagnosis, while highly increasing assay accuracy and lowering detection limit. Moreover, this PCR-based method is relatively simple and inexpensive, and it does not require the time-consuming step of pre-incubation on microbiological media (Schaad et al. 1995). Even in the presence of potential inhibitors present in plant material, which can affect the limit of detection, we could detect 1 and 3 × 10^2^ cfu/reaction using primers specific for *Psm*1 and *Psm*2 in conventional PCR. A similar detection sensitivity in conventional PCR was obtained by other authors in their identification systems for other phytopathogens (Catara et al. 2000; Kerkoud et al. 2002; Biondi et al. 2013). The sensitivity of real-time PCR was higher than in the case of conventional ones, as 1 cfu/reaction was detected when different templates were used. This is especially important in the case of naturally infected material in the presence of a small amount of pathogen DNA, which be detected in a very short time. The limit of detection when using gDNA was in the range from ~4–5 pg in conventional and ~10–100 fg in real-time PCR for both taxa, which are similar to results obtained for *P. syringae* pv. *actinidiae* (Gallelli et al. 2014) and *Clavibacter michiganensis* subsp. *sepedonicus* (Cho et al. 2015). The high sensitivity of the developed assay (obtained in our hands) will be invaluable for detecting the target bacteria in the early latent period of the disease, allowing growers to undertake appropriate prevention or protection programmes.

## References

[CR1] Agrios GN (2005) Plant diseases caused by prokaryotes: bacteria and mollicutes, chapter 12. In: Agrios GN (ed) Plant Pathology, 5th edn. Elsevier Academic Press, San Diego, USA, p 616–703

[CR2] Ajmone-Marsan P, Valentini A, Cassandro M, Vecchiotti-Antaldi G, Bertoni G, Kuijper M (1997). AFLP markers for DNA fingerprinting in cattle. Anim Genet.

[CR3] Albuquerque P, Caridade CMR, Marcal ARS, Cruz J, Cruz L, Santos CL, Mendes MV, Tavares F (2011). Novel markers for identification of *Xanthomonas fragariae*, *Xanthomonas axonopodis* pv. *phaseoli* and *Xanthomonas fuscans* subsp. *fuscans* using a dot blot platform coupled with automatic data analysis. Appl Environ Microbiol.

[CR4] Aljanabi SM, Martinez I (1997). Universal and rapid salt extraction of high quality genomic DNA for PCR-based techniques. Nucl Acids Res.

[CR5] Altschul SF, Madden TL, Schaffer AA, Zhang J, Zhang Z, Miller W, Lipman DJ (1997). Gapped BLAST and PSI-BLAST: a new generation of protein database search programs. Nucl Acids Res.

[CR6] Bereswill S, Bugert P, Volksch B, Ullrich M, Bender CL, Geider K (1994). Identification and relatedness of coronatine-producing *Pseudomonas syringae* pathovars by PCR analysis and sequence determination of the amplification products. Appl Environ Microbiol.

[CR7] Biondi E, Galeone A, Kuzmanovic N, Ardizzi S, Lucchese C, Bertaccini A (2013). *Pseudomonas syringae* pv. *actinidiae* detection in kiwifruit plant tissue and bleeding sap. Ann Appl Biol.

[CR8] Bull CT, Manceau C, Lydon J, Kong H, Vinatzer BA, Fischer-Le Saux M (2010). *Pseudomonas cannabina* pv. *cannabina* pv. nov., and *Pseudomonas cannabina* pv. *alisalensis* (Cintas Koike and Bull, 2000) comb. nov., are members of the emended species *Pseudomonas cannabina* (ex Sutic & Dowson 1959) Gardan, Shafik, Belouin, Brosch, Grimont & Grimont 1999. Syst Appl Microbiol.

[CR9] Bull CT, Clarke CR, Cai R, Vinatzer BA, Jardini TM, Koike ST (2011). Multilocus sequence typing of *Pseudomonas syringae* sensu lato confirms previously described genomospecies and permits rapid identification of *P. syringae* pv. *coriandricola* and *P. syringae* pv. *apii* causing bacterial leaf spot on parsley. Phytopathology.

[CR10] Bultreys A, Gheysen I (1999). Biological and molecular detection of toxic lipodepsipeptide-producing *Pseudomonas syringae* strains and PCR identification in plants. Appl Environ Microbiol.

[CR11] Bultreys A, Kałużna M (2010). Bacterial cankers caused by *Pseudomonas syringae* on stone fruit species with special emphasis on the pathovars *syringae* and *morsprunorum* race 1 and race 2. J Plant Pathol.

[CR12] Bultreys A, Gheysen I, de Hoffmann E (2006). Yersiniabactin production by *Pseudomonas syringae* and *Escherichia coli* and description of a second yersiniabactin locus evolutionary group. Appl Environ Microbiol.

[CR13] Catara V, Arnold D, Cirvilleri G, Vivian A (2000). Specific oligonucleotide primers for the rapid identification and detection of the agent of tomato pith necrosis, *Pseudomonas corrugata*, by PCR amplification: evidence for two distinct genomic groups. Eur J Plant Pathol.

[CR14] Cheng J, Long Y, Khan MA, Wei C, Fu S, Fu J (2015). Development and significance of RAPD-SCAR markers for the identification of *Litchi chinensis* Sonn. by improved RAPD amplification and molecular cloning. Electron J Biotechnol.

[CR15] Cho MS, Park DH, Namgung M, Ahn T-Y, Park DS (2015). Validation and application of a real-time PCR protocol for the specific detection and quantification of *Clavibacter michiganensis* subsp. *sepedonicus* in potato. Plant Pathol J.

[CR16] Gallelli A, Talocci S, Pilotti M, Loreti S (2014). Real-time and qualitative PCR for detecting *Pseudomonas syringae* pv. *actinidiae* isolates causing recent outbreaks of kiwifruit bacterial canker. Plant Pathol.

[CR17] Gardan L, Shafik H, Belouin S, Broch R, Grimont F, Grimont PAD (1999). DNA relatedness among the pathovars of *Pseudomonas syringae* and description of *Pseudomonas tremae* sp. nov. and *Pseudomonas cannabina* sp. nov. (ex Sutic and Dowson 1959). Int J Syst Bacteriol.

[CR18] Giaj Merlera G, Muñoz S, Coelho I, Cavaglieri LR, Torres AM, Reynoso MM (2015). Diversity of black Aspergilli isolated from raisins in Argentina: polyphasic approach to species identification and development of SCAR markers for *Aspergillus ibericus*. Int J Food Microbiol.

[CR19] Gilbert V, Legros F, Maraite H, Bultreys A (2009). Genetic analyses of *Pseudomonas syringae* isolates from Belgian fruit orchards reveal genetic variability and isolate-host relationships within the pathovar *syringae*, and help identify both races of the pathovar *morsprunorum*. Eur J Plant Pathol.

[CR20] Kałużna M, Ferrante P, Sobiczewski P, Scortichini M (2010). Characterization and genetic diversity of *Pseudomonas syringae* isolates from stone fruits and hazelnut using repetitive-PCR and MLST. J Plant Pathol.

[CR21] Kałużna M, Puławska J, Sobiczewski P (2010). The use of PCR melting profile for typing of *Pseudomonas syringae* isolates from stone fruit trees. Eur J Plant Pathol.

[CR22] Kałużna M, Janse JD, Young JM (2012). Detection and identification methods and new tests as used and developed in the framework of COST 873 for bacteria pathogenic to stone fruits and nuts *Pseudomonas syringae* pathovars. J Plant Pathol.

[CR23] Kałużna M, Puławska J, Waleron M, Sobiczewski P (2014). The genetic characterization of *Xanthomonas arboricola* pv. *juglandis*, the causal agent of walnut blight in Poland. Plant Pathol.

[CR24] Kerkoud M, Manceau C, Paulin JP (2002). Rapid diagnosis of *Pseudomonas syringae* pv. *papulans*, the causal agent of blister spot of apple, by polymerase chain reaction using specifically designed *hrpL* gene primers. Phytopathology.

[CR25] King EO, Raney MK, Ward DE (1954). Two simple media for the demonstration of pyocianin and fluorescin. J Lab Clin Med.

[CR26] Lattore BA, Jones AL (1979). *Pseudomonas morsprunorum*, the cause of bacterial canker of sour cherry in Michigan and its epiphytic association with *P. syringae*. Phytopathology.

[CR27] Leibner-Ciszak J, Dobrowolska A, Krawczyk B, Kaszuba A, Staczek P (2010). Evaluation of a PCR melting profile method for intraspecies differentiation of *Trichophyton rubrum* and *Trichophyton interdigitale*. J Med Microbiol.

[CR28] Lelliott RA, Billing E, Hayward AC (1966). A determinative scheme for the fluorescent plant pathogenic Pseudomonads. J Appl Bacteriol.

[CR29] Lim SH, Kim JG, Kang HW (2009). Novel SCAR primers for specific and sensitive detection of *Agrobacterium vitis* strains. Microbiol Res.

[CR30] Liu Y, Li S, Zhu T, Shao B (2012). Specific DNA markers for detection of bacterial canker of kiwifruit in Sichuan, China. Afr J Microbiol Res.

[CR31] López M, Roselló M, Palacio-Bielsa A (2010). Diagnosis and detection of the main bacterial pathogens of stone fruit and almond. J Plant Pathol.

[CR32] Masny A, Płucienniczak A (2003). Ligation mediated PCR performed at low denaturation temperatures-PCR melting profiles. Nucl Acids Res.

[CR33] Ménard M, Sutra L, Luisetti J, Prunier JP, Gardan L (2003). *Pseudomonas syringae* pv. *avii* (pv. nov.), the causal agent of bacterial canker of wild cherries (*Prunus avium*) in France. Eur J Plant Pathol.

[CR34] Puławska J, Maes M, Deckers T, Sobiczewski P (1997). The influence of pesticide contamination on detection of epiphytic *Erwinia amylovora* using PCR. Meded Fac Landbouwkd Toegep Biol Wet Univ Gent.

[CR35] Ramakers C, Ruijter JM, Deprez RHL, Moorman AFM (2003). Assumption-free analysis of quantitative real-time polymerase chain reaction (PCR) data. Neurosci Lett.

[CR36] Renick LJ, Cogal AG, Sundin GW (2008). Phenotypic and genetic analysis of epiphytic *Pseudomonas syringae* populations from sweet cherry in Michigan. Plant Dis.

[CR37] Sangdee A, Natphosuk S, Srisathan A, Sangdee K (2013). Development of SCAR primers based on a repetitive DNA fingerprint for *Escherichia coli* detection. J Microbiol.

[CR38] Saniewski M, Ueda J, Miyamoto K, Horbowicz M, Puchalski J (2006). Hormonal control of gummosis in *Rosaceae*. J Fruit Ornam Plant Res.

[CR39] Schaad NW, Cheong SS, Tamaki E, Hatziloukas E, Panopoulos NJ (1995). A combined biological and enzymatic amplification (BIOPCR) technique to detect *Pseudomonas syringae* pv. *phaseolicola* in bean seed extracts. Phytopathology.

[CR40] Schmidt O, Dujesiefken D, Stobbe H, Moreth U, Kehr R, Schröder T (2008). *Pseudomonas syringae* pv. *aesculi* associated with horse chestnut bleeding canker in Germany. For Pathol.

[CR41] Sorensen KN, Kim K-H, Takemoto JY (1998). PCR Detection of cyclic lipodepsinonapeptide-producing *Pseudomonas syringae* pv. *syringae* and similarity of strains. Appl Environ Microbiol.

[CR42] Suslow TV, Schrooth MN, Isaka M (1982). Application of a rapid method for Gram differentiation of plant pathogenic and saprophytic bacteria without staining. Phytopathology.

[CR43] Ullrich M, Bereswill S, Volksch B, Fritsche W, Geider K (1993). Molecular characterization of field isolates of *Pseudomonas syringae* pv. *syringae* differing in coronatine production. J Gen Microbiol.

[CR44] Vicente JG, Roberts SJ (2007). Discrimination of *Pseudomonas syringae* isolates from sweet and wild cherry using rep-PCR. Eur J Plant Pathol.

[CR45] Vicente JG, Alves JP, Russell K, Roberts SJ (2004). Identification and discrimination of isolates from wild cherry in England. Eur J Plant Pathol.

[CR46] Waugh R, Bonar N, Baird E, Thomas B, Graner A, Hayes P, Powell W (1997). Homology of AFLP products in three mapping populations of barley. Mol Gen Genet.

[CR47] Weingart H, Völksch B (1997). Genetic fingerprinting of *Pseudomonas syringae* pathovars using ERIC-, REP-, and IS50-PCR. J Phytopathol.

[CR48] Young JM (2010). Taxonomy of *Pseudomonas syringae*. J Plant Pathol.

[CR49] Zasada AA, Formińska K, Wołkowicz T, Badell E, Guiso N (2014). The utility of the PCR melting profile technique for typing *Corynebacterium diphtheriae* isolates. Lett Appl Microbiol.

[CR50] Zhang M, Chen WQ, Liu D, Liu TG, Gao L, Shu K (2012). Identification of a specific SCAR marker for detection of *Tilletia foetida* (Wall) Liro pathogen of wheat. Russ J Genet.

